# Determination of the dynamic cellular transcriptional profiles during kidney development from birth to maturity in rats by single-cell RNA sequencing

**DOI:** 10.1038/s41420-021-00542-9

**Published:** 2021-06-24

**Authors:** Fangrui Ding, Xiuying Tian, Jiali Mo, Botao Wang, Jun Zheng

**Affiliations:** 1 Department of Neonatology, Tianjin Central Hospital of Obstetrics and Gynecology, Tianjin, China; 2 Tianjin Key Laboratory of Human Development and Reproductive Regulation, Tianjin, China; 3 Department of Neonatology, Nankai University Maternity Hospital, Tianjin, China; 4 Graduate School, Tianjin Medical University, Tianjin, China

**Keywords:** Developmental biology, Cell biology

## Abstract

Recent single-cell RNA sequencing (scRNA-seq) analyses have offered much insight into the gene expression profiles in early-stage kidney development. However, comprehensive gene expression profiles from mid- and late-stage kidney development are lacking. In the present study, by using the scRNA-seq technique, we analyzed 54,704 rat kidney cells from just after birth to adulthood (six time points: postnatal days 0, 2, 5, 10, 20, and 56) including the mid and late stages of kidney development. Twenty-five original clusters and 13 different cell types were identified during these stages. Gene expression in these 13 cell types was mapped, and single cell atlas of the rat kidney from birth to maturity (http://youngbearlab.com) was built to enable users to search for a gene of interest and to evaluate its expression in different cells. The variation trend of six major types of kidney cells—intercalated cells of the collecting duct (CD-ICs), principal cells of the collecting duct (CD-PCs), cells of the distal convoluted tubules (DCTs), cells of the loop of Henle (LOH), podocytes (PDs), and cells of the proximal tubules (PTs)—during six postnatal time points was demonstrated. The trajectory of rat kidney development and the order of induction of the six major types of kidney cells from just after birth to maturity were determined. In addition, features of the dynamically changing genes as well as transcription factors during postnatal rat kidney development were identified. The present study provides a resource for achieving a deep understanding of the molecular basis of and regulatory events in the mid and late stages of kidney development.

## Introduction

Prematurity is the most common cause of hospitalization in neonatal intensive care units (NICUs) [1, 2]. The major issue in preterm infants is the immaturity of organs and systems at birth and then abnormal developmental programs after birth when compared with full term infants, which strongly increase the risk of various long-term chronic diseases. This hypothesis is also called the developmental origins of health and disease (DOHaD), which proposes that deleterious events occurring in the earliest stages of human development affect the long term occurrence of diabetes, cardiovascular disease, asthma, neuropsychiatric disorders, chronic kidney disease (CKD), and so on [3–5]. In the field of kidney disease, Brenner et al. first proposed that a congenital decrease in nephron number could cause renal injury, which is similar to the DOHaD hypothesis [6, 7]. This hypothesis provides a plausible explanation for the high prevalence of renal disease in low birth weight or preterm infants, who are expected to show a low number of nephrons [6, 7]. Currently, an increasing number of clinical studies have shown that prematurity is a risk factor for CKD [8–12]. However, the underlying molecular mechanism has not been elucidated.

Most preterm infants are born at 21 to 37 weeks of gestation corresponding to mid and late gestation [13, 14]. Before kidney development in preterm infants can be fully elucidated, the normal physiological gene expression patterns during this age should be determined. In recent years, single-cell RNA sequencing (scRNA-seq) has made a major contribution to elucidating developmental processes as well as mapping gene profiles during organ development, including kidney development [15–25]. Many studies have provided comprehensive and dynamic gene expression profiles during kidney development by using scRNA-seq [16, 18, 20–22]. However, most of these studies focused on embryonic kidney development and early fetal kidney development (corresponding to less than 20 gestational weeks). Few studies have focused on the mid and late stages of kidney development. Because it is difficult to acquire kidney samples during 21–37 gestational weeks in humans, murine can be used as a good model for elucidating the development molecular mechanism of this organ during this age.

Kidney development between murine and humans is different. In humans, nephrogenesis (the formation of nephrons) starts at 4–5 weeks of gestation and is completed in utero at gestational week 36 before full-term birth. In mice and rats, nephrogenesis begins on embryonic days 11 and 12, respectively, and is completed at ~20 days after birth. Therefore, the difference between humans and murine is that nephrogenesis in humans is completed in utero before birth, while the murine kidney is still immature at birth, and nephrogenesis has both intra- and extra-uterine developmental stages [26–29]. The extra-uterine developmental stage of the murine kidney corresponds to mid and late gestational kidney development in humans. Because of this characteristic of extra-uterine development in mice and rats, acquiring kidney samples for further study is very convenient during this stage, and instead of calculating the precise embryonic age, researchers can wait for murine birth. Thus, a postnatal murine model could be the best model for determining the mid and late gestational development of the kidney. In the present study, a scRNA-seq of 54,704 renal cells spanning postnatal day 0–postnatal day 56 (corresponding to mid, late, and mature stages of kidney development) was performed. Distinct cell populations, as well as gene expression profiles, were identified, and developmental trajectories of the major kidney cell populations were determined in this study. In addition, due to the limited amount of published gene expression data from the mid to late stages of renal development in any species, the sequence dataset has also been presented as a database at http://youngbearlab.com; this database is being provided as a resource for individual researchers to perform their own sophisticated analyses of questions of interest.

## Results

### Clustering of cells during postnatal kidney development of rats

**Fig. 1:**
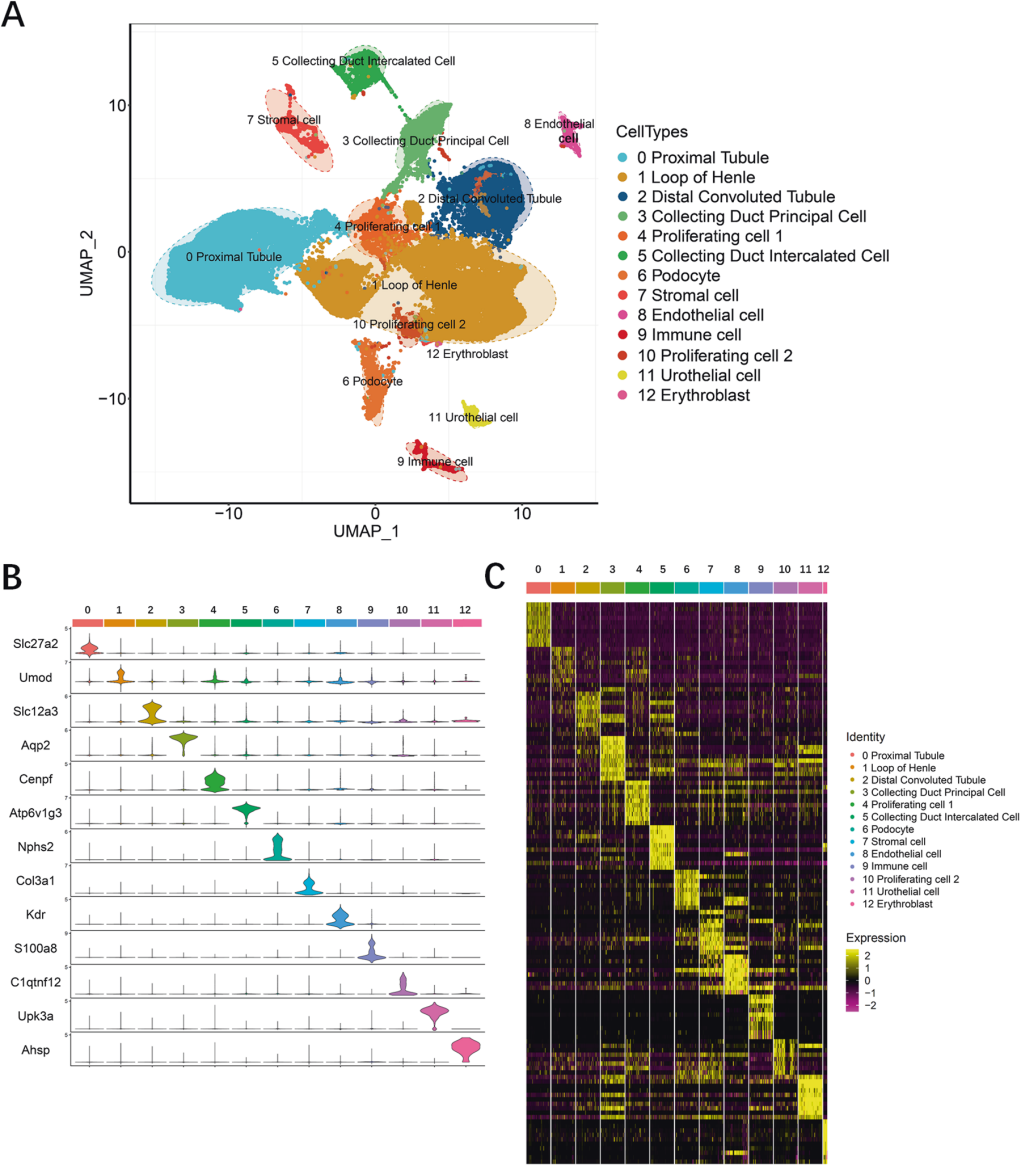
Cell types during the development of the postnatal rat kidney delineated by single-cell transcriptomic analysis. **A** Postnatal rat kidneys were collected at days 0, 2, 5, 10, 20, and 56 after birth, and after preparation, single-cell suspension sequencing and analysis were performed. Thirteen distinct cell clusters revealed by unsupervised clustering and shown in a two-dimensional UMAP map. A total of 54,704 cells and 25 original clusters were identified. After the analysis of differentially expressed genes in the 25 original clusters with known gene markers, 13 different cell types were identified and annotated, as shown on the right side of UMAP results. **B** Violin plots showing the expression level of representative marker genes across the 13 clusters. The vertical axis shows the gene expression value. Each color represents one cluster as indicated on the right side in **C**. The annotations of each color are presented on the right side of **C**. **C** Heatmap of the gene expression patterns of the top ten cluster-specific genes in 13 clusters. Different clusters are identified by different colors, as indicated at the top of the heatmap and annotated on the right side of the heatmap. The color bar ranging from yellow to red-purple reflects the relative expression levels from high to low.

In the present study, six-time points were chosen for mapping the gene expression of postnatal kidney development in rats: postnatal days 0, 2, 5, 10, 20, and 56, which correspond to kidney development stages in most human preterm infants according to pathological findings. During postnatal day 0–postnatal day 5, nephrogenesis was clearly detected. At this age, the comma- and S-shaped bodies were detected in the outer renal cortex. During postnatal day 10–postnatal day 20, obvious nephrogenesis was not detected in the kidney, while glomeruli were still immature. After postnatal day 20, most of the glomeruli, as well as the tubules, were mature [28–32]. To identify the gene expression profiles during these times, we performed single-cell sequencing and analysis. Finally, 54,704 cells were obtained, and uniform manifold approximation and projection (UMAP) analysis of the combined datasets yielded 25 original clusters, and the median expression levels of each gene in each cluster are provided in Supplement Table 1. According to the differentially expressed genes (DEGs) in the 25 original clusters (Supplement Table 2) and annotation by known gene markers, 13 different cell types were identified overall (Fig. 1A and Fig. S1).

### Cellular diversity of rat kidney development after birth identified by single-cell transcriptomic analysis

To define the identity of each cluster, the expression patterns of previously identified cell-type-specific markers in each cell cluster were examined. As shown in Fig. 1, cluster 0 represented proximal tubules (PTs), as these cells highly expressed the specific PT genes SLC27A2 and CUBN [33, 34]. Cluster 1 highly expressed UMOD and SLC12A1 and belonged to the loop of Henle (LOH) [35, 36]. Cluster 2 represented distal convoluted tubules (DCTs), as they highly expressed SLC12A3 [37]. Cluster 3 highly expressed principal cells of the collecting duct (CD-PCs) marker genes, such as AQP2 and AQP3 [38, 39]. In cluster 4, the proliferating cell 1 (PC1) marker genes TOP2A and CENPF were highly expressed [18]. For the identification of intercalated cells of the collecting duct (CD-ICs), ATP6V1G3 and ATP6V0D2 were selected, and cluster 5 was identified [17]. Podocytes (PDs) highly express NPHS1 and NPHS2, and cluster 6 was identified [40, 41]. Stromal cells (SCs) highly express COL3A1 and LGALS1, which were identified in cluster 7 [42, 43]. Endothelial cells (ECs) are represented by cluster 8, which highly expressed KDR and PLVAP [44, 45]. Cluster 9 highly expressed S100A8 and S100A9 [46]; thus, this cluster represents immune cells (ICs). Cluster 11 represented a small group of urothelial cells (UCs), as these cells specifically express UPK3A [47]. HBB, ALAS2, and AHSP were highly expressed in cluster 12, namely, erythroblasts (ERs) [48, 49].

There are three clusters that were difficult to annotate: original clusters 1, 20, and 21 (Supplement Table 2 and Fig. S1). For clusters 1 and 20, highly DEGs were CRYAB, TSPAN8, SPP1, IGFBP2, and SLC31A2. After annotation by several kidney studies and kidney databases such as GenitoUrinary Development Molecular Anatomy Project (GUDMAP) (This database contains a molecular atlas of gene expression for the developing organs of the GenitoUrinary tract and addresses at https://www.gudmap.org/) and the Human Protein Atlas (HPA) (this database contains the distribution of the proteins across all major tissues and organs in the human body and expression of protein-coding genes in a single human cell type and addresses at https://www.proteinatlas.org/), we found that these genes are not limited to one known cell type [16–19, 22, 23]. They have been found to be expressed in tubules (LOH, PTs, DCTs, and CD-PCs). Then, we performed subclustering of these two clusters, as shown in Supplement Table 3. These cells were clustered into nine subclusters. According to studies to identify gene profiles of the separate kidney zones by Andrew Ransick et al. and Jae Wook Lee [19, 50], the top DEGs (TSPAN8, ID1, ID3, and JAG1) in subcluster 0 and subcluster 1 were highly expressed in short descending limbs of LOH. The top DEGs (CLDN10, BSND, and CLCNKA) in subcluster 2 were highly expressed in thin ascending limbs of LOH. The top DEGs (EGR1, FOS, and EHF) in subcluster 3 and subcluster 6 were highly expressed in thin descending limbs of LOH. Additionally, the top DEGs were also assessed with Kidney Cell Explorer (This explorer is a database of kidney single-cell database) [19]. As a result, most of these genes were highly expressed in the LOH cell population (Fig. S2). Thus, original clusters 1 and 20 were annotated as the LOH.

It was also difficult to annotate original cluster 21 (present cluster 10). We examined the expression pattern of the top DEGs such as HAS2 and WNT4 in all cells and viewed the results in UMAP. Most of these genes were expressed in proliferating cells (original cluster 11, present cluster 4) (Fig. S3A–D). In addition, based on cluster hierarchy (Build Cluster Tree function) (Fig. S3E), these two clusters originated from the same hierarchy. Then, this cluster was a highly likely proliferating cell. However, the marker genes TOP2A and CENPF highly expressed in cluster 4 were not found in this cluster; therefore, this cluster was annotated as proliferating cell 2 (PC2).

After annotation all clusters, we have also built a searchable database including a single-cell atlas of the rat kidney from birth to maturity (http://youngbearlab.com). Users can search for a gene of interest and evaluate its expression in different cells.

### Distinct phases of kidney development after birth

**Fig. 2:**
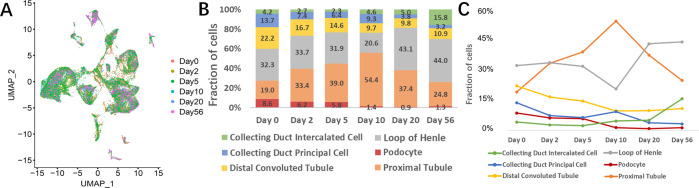
Dynamic changes in major kidney cell types over time. **A** UMAP plots of six different developmental stages of rat kidney after birth. **B** The six major kidney cell types, namely, collecting duct intercalated cell, collecting duct principal cell, distal convoluted tubule, loop of Henle, podocyte, and proximal tubule were normalized to 100% at the same time point. The proportions of each cell type at each point are stacked, and dynamic changes are presented over development time. **C** The change trends of the proportion of each major kidney cell type during different developmental stages are indicated with broken lines. Podocyte, collecting duct principal cells, and distal convoluted tubules showed decreases during the postnatal period while collecting duct principal cells show an increase over time. Loop of Henle showed a decrease from postnatal days 0 to 10 and then an increase, while proximal tubules present the opposite pattern.

For analysis of the dynamic changes in the cell population at different times, all the cells from six separate time points are shown in the UMAP graph (Fig. 2A). Total six major kidney cell populations, including CD-IC, CD-PC, DCT, LOH, PD, and PT have been normalized to 100% at the same time point and then each cell type proportion has been recalculated and presented in Fig. 2B. The changing trends of each cell type during the time were combined and presented in Fig. 2C. As shown in Fig. 2B, C, the numbers of PDs, CD-PCs, and DCTs decreased during the postnatal period, while that of CD-ICs increased over time. The general trend of the percentage of the LOH was to decrease first and then to increase. The percentage of PT gradually increased from postnatal day 0 to postnatal day 10. After postnatal day 10, it decreased.

### Pseudotemporal ordering of major kidney cells during postnatal kidney development

**Fig. 3:**
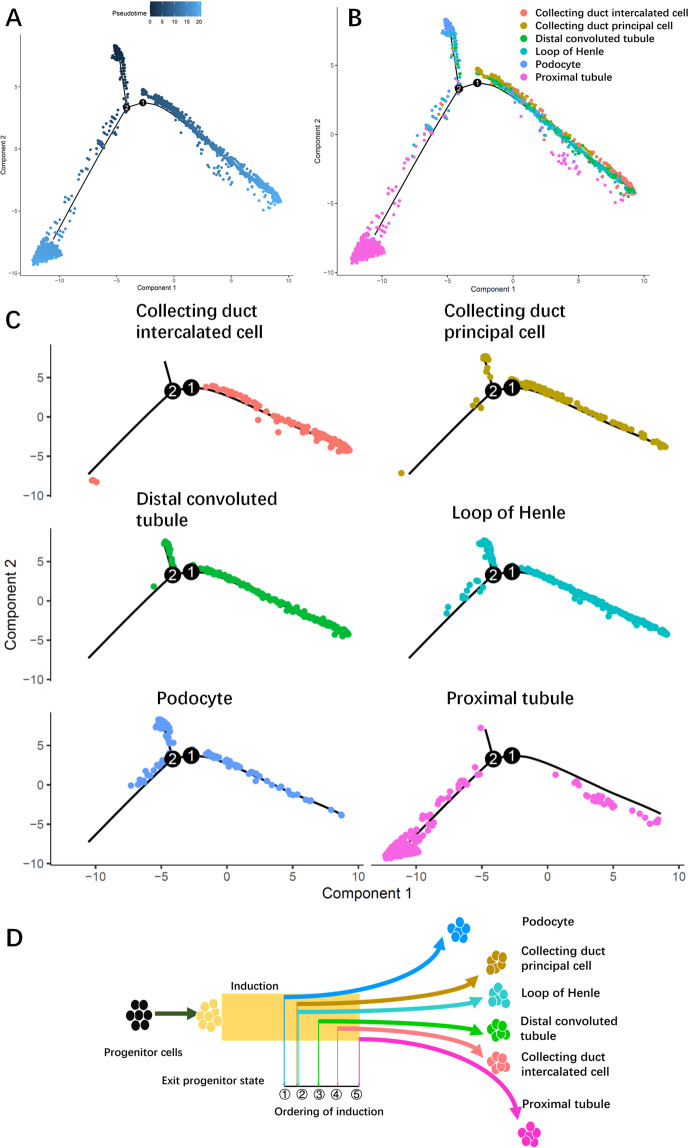
Distinct phases of major kidney cell clusters during the development of postnatal rat kidney. **A**, **B** Pseudotime trajectory of the major kidney cells (collecting duct intercalated cells, collecting duct principal cells, distal convoluted tubules, loop of Henle, podocytes, and proximal tubules). Cells are colored based on the predicted pseudotime. The deeper the color, the earlier it is (**A**). Cell type transition during the development of rat kidney after birth according to pseudotime path (**B**). **C** Split monocle pseudotime plot revealed detailed development of the major kidney cell clusters. **D** Overview of different cell cluster fates along cumulative time as indicated by pseudotime. Numbers 1–5 present the ordering of cell type induction based on pseudotime. Podocytes were the first cell type to be induced from the progenitors and to reach their mature fate, followed by the loop of Henle and collecting duct principal cells, and then distal convoluted tubules and collecting duct intercalated cells, with proximal tubules being the final mature cell type observed kidney development.

Previously, single-cell sequence analyses have revealed that the early stage of kidney development corresponds to the early gestational weeks [16, 18–20, 22]. To elucidate gene expression dynamics during postnatal to mature kidney development, we performed pseudotemporal ordering of the major kidney cells (PD, PT, LOH, DCT, CD-PC, and CD-IC) in the present study. As shown in Fig. 3A, B, the initiation period was not limited to one or two cell populations because of the period of time of samples we chose. This period contained PD, LOH, DCT, and CD-PC. There are two major paths during postnatal rat kidney development. One involves CDs, LOHs, and DCTs. Another involves PTs. As shown in Fig. 3C, the split trajectories of each cell type showed that PD was the first cell type presented and fated to mature, followed by LOH and CD-PC, which were ordered by DCT and CD-IC. Finally, PT was induced to mature during kidney development. Previous study has shown that nephron progenitor cells are induced to two major distinct trajectories: PDs and proximal/distal tubule fates [21, 22]. In those studies, PDs are induced from progenitor cells earlier than tubules [21, 22], which is same with our founding. In detail, the accurate order was determined in this study when combined with previous studies describing the state from progenitor cells to maturity (Fig. 3D).

### Features of dynamically changing genes during postnatal rat kidney development

**Fig. 4:**
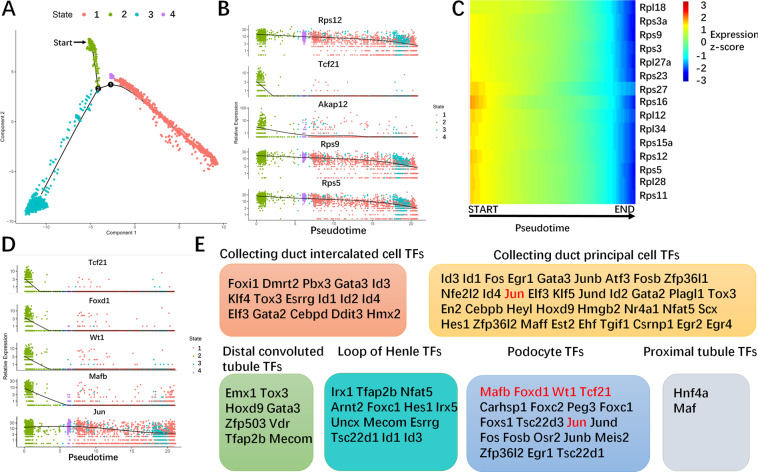
Features of dynamically changing genes over temporal trajectories. **A** Cell state transition according to the pseudotime path. There are four major states over pseudotime. **B** Expression patterns of the top five DEGs over pseudotime. **C** Heatmap of ribosomal protein genes in the top 50 DEGs of the pseudotime path are significantly changed in the pseudotime path. All of these genes were highly expressed at the earlier stage and decreased during time. **D** Top five TFs in the top 200 DEGs over the pseudotime are listed and the expression patterns of these TFs over pseudotime were mapped. **E** TFs enriched in the six major kidney cell types are listed. The genes highlighted in red in each box were top five TFs among the top 200 DEGs over the pseudotime. All of the top five TFs were enriched in podocytes indicating the important role of podocytes during these development stages. DEGs differentially expressed genes, TFs transcriptional factors.

To further map the gene expression dynamics and to find potential key genes of signaling pathways during postnatal kidney development, we analyzed DEGs during postnatal kidney development along the pseudotime. First, the top five DEGs in pseudotime were identified, and 3/5 were genes that encode ribosomal proteins and related proteins (Fig. 4A, B). The percentage was unexpectedly high. Then, the top 50 DEGs in pseudotime were identified (Supplement Table 4). These genes accounted for 30% (15/50) of the DEGs. As shown in Fig. 4B, C, at the start of postnatal kidney development in rats, these genes were highly expressed and gradually decreased during pseudotime. Ribosomal proteins are known to be involved in protein synthesis [51–53]. The dynamic changes in these genes suggested that high levels of proteins were produced during the mid and late stages of kidney development. In addition to ribosomal proteins, the top 200 DEGs of each major kidney cell type (CD-IC, CD-PC, DCT, LOH, PD, and PT) (Supplement Table 5) except ribosomal proteins from pseudotemporal ordering analysis were chosen by using GO analysis. As shown in Fig. S4, in the GO analysis, most of the GO terms were involved in translational and posttranslational processes including proteasome complex, methylosome, and chaperone complex in the cellular component module (Fig. S4) and translation factor activity, RNA binding, unfolded protein binding, and ubiquitin protein ligase binding in the molecular function module (Fig. S4 and Supplement Table 6), which suggested that different cell types complete their fate and then initiate a large number of functional unit synthesis of each lineage during mid and late stages of kidney development [16, 21]. In addition, DNA-binding transcriptional factors (TFs) can play roles in translational regulation during development. Figure 4D lists the top five TFs among the top 200 DEGs over pseudotime. They include TCF21, FOXD1, WT1, MAFB, and JUN. Moreover, the TFs enriched in six major kidney cell types are also listed in Fig. 4E.

#### Cell-type-specific expression of CKD-related genes

**Fig. 5:**
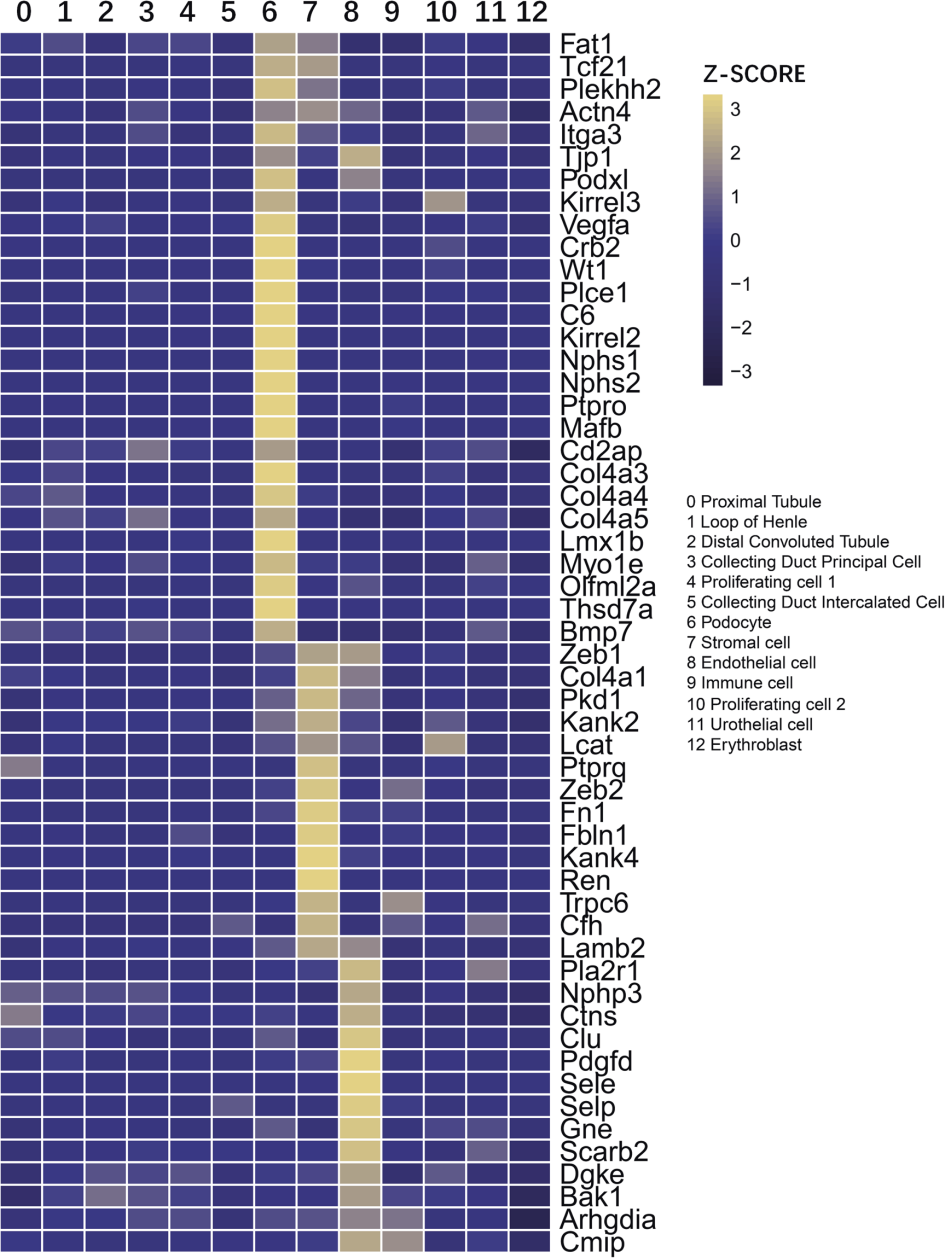
Expression features of known glomerular diseases genes. Clustered expression of known glomerular disease genes collected from OMIM databases and genes showing high expression in podocytes, endothelial cells, and stroma cells are listed. Color keys indicate relative expression level and based on the *z*-score distribution. In the heatmap, each row represents one gene and each column is a single cell type. The full list of known glomerular disease genes and cell type is shown in Fig. S5.

Prematurity is a risk factor for CKD [8–12]. Though there are many possible causes of CKD, the major cause is glomerular diseases [54]. The known glomerular disease genes were collected from the OMIM database, and the expression features of these genes were examined during these stages. There are three major cell types in the glomerulus: PDs, ECs, and mesangial cells. In the present study, intraglomerular ECs and mesangial cells were not separated from total ECs and stroma cells. Hence, these genes were examined in PDs, ECs, and stroma cells. As shown in Figs. 5, S5, most of these genes were highly expressed in these three types of cells, among which PDs accounted for the largest percentage.

## Discussion

In humans, normal nephrogenesis is completed in utero in 36 weeks, indicating that most preterm infants experience a potential abnormal extra-utero developmental stage [26]. The gestational weeks of most preterm infants range from 21 to 37 weeks [13, 14], which corresponds to the mid and late developmental stages of the kidney. Many studies have focused on embryonic kidney development, early fetal kidney development, or mapping the gene expression profile of the mature kidney. In a study by Lindstrom et al., researchers clearly identified a time-dependent process of cell fate in early nephrogenesis [21]. In a study by Wang et al., early fetal kidney development was explored [16]. In addition, Park et al. mapped mature kidney gene expression patterns [17]. However, few studies have explored the mid and late stages of kidney development to maturity. To our knowledge, the present study may be the first to reveal the dynamic physiological gene expression profiles of the rat kidney from birth to maturity based on scRNA-seq analysis. In addition, the sequence data from the present study will serve as a resource for generating an atlas of gene expression during these stages. Considering the limited knowledge about all aspects of kidney development described herein, other researchers will be most welcome to use our data to reveal more details during these stages. To ensure that our sequence data are available to every potential user, we have generated a web database to facilitate access to our data and analysis of these data by the academic and clinical research communities. Users can search for the gene of interest and evaluate the expression in different cells. Additionally, this resource was produced in rat; in the future, it is expected that human data during the mid- and late developmental stages will be made available and that comparisons of rat and human data will be performed to explore the differences between these two species [55].

During the mid and late stages of kidney development, the development of the LOH is one of the major developmental processes [18, 19, 56, 57]. In the present study, the LOH exhibited the largest number of cells (19,651/54,704 = 35.9%), which confirmed the possible key role during mid and late kidney development. Thus, negative factors occurring during the mid and late stages of kidney development may also cause an adverse effect on the LOH. The LOH plays a role in successful radiation, and short loop nephrons have been associated with smaller glomeruli [56]. Previous studies have shown that preterm individuals have a reduced nephron number and increased number of abnormal glomeruli [6, 7]. Though little is known in preterm infants regarding whether the LOH affects abnormal glomeruli, we still considered that LOH development plays an important role in preterm kidney developmental processes. In addition, experts on the LOH are encouraged to access our database for further research.

In addition to the gene expression profile presented in this study, the time development process of different kidney cell types was shown. Furthermore, the DEGs during pseudotime were examined. In the top 50 DEGs during pseudotime, genes that encode ribosomal proteins accounted for a large percent. Ribosomes are organelles for protein synthesis [51–53]. A large number of ribosomal protein-related genes have been highlighted, which suggests that high protein levels are produced at the early stage of postnatal development compared with the mature stage. In addition to ribosomal proteins, GO analyses except for ribosomal proteins from pseudotemporal ordering analysis were also performed. Most of the GO terms were involved in translational and posttranslational processes (Fig. S4 and Supplement Table 6). These results indicated the importance of protein synthesis and protein modification and correspond to the formation of a larger number of functional units and organ size enlargement during the mid and late stages of development [58, 59]. RPS12 is one of ribosomal related protein, which is also the top DEG during pseudotime in the present study. Though the role of this gene in kidney development has not been examined, a study in *Drosophila* strongly suggested that RPS12 is a determinant for organ growth and size in development [60]. In preterm infants, the functional unit nephrons were found in reduced numbers [6, 7] and further experimental studies should examine whether ribosomal proteins have an effect on nephron number and kidney size in preterm infants.

The TFs among the top 200 DEGs during pseudotime were listed. FOXD1, one of top five TFs among the top 200 DEGs over pseudotime, has already been shown to be a specific marker in stromal progenitor cells and is essential for normal glomerular development [61, 62]. In addition, MAFB is required for the differentiation of glomerular visceral epithelial cells during kidney development [63]. TCF21, another top five TF, was the first tissue-restricted basic helix–loop–helix protein identified in the developing kidney, and it plays a role in the regulation of morphogenetic events during kidney development [64]. WT1 has been confirmed to play an important role not only in kidney development but also in the pathogenesis of kidney diseases [65]. In addition, JUN has been reported to be involved in cell proliferation during kidney development [66]. All of the top five TFs among the top 200 DEGs over pseudotime play key roles in kidney development, which indicated that our study has a potential role in identifying or predicting important molecular factors involved in kidney development. Thus, the remaining differentially expressed TFs presented in our study could be the candidate key TF during the mid and late stages of kidney development. We have already listed these TFs in six major kidney cell types. These TFs are resources included in our database. Further experimental studies could also focus on these candidate TFs.

## Materials and Methods

### Animals

Animal experiments were approved by the Tianjin Central Hospital of Gynecology and Obstetrics Animal Ethics Committee. All animal experimental protocols conformed to the Institutional Animal Care Guidelines. Pregnant Sprague-Dawley rats were purchased from Charles River Laboratories (Beijing, China). At each time point, three rats (pups) at each time point were anesthetized by CO_2_ inhalation, and then their kidneys were pooled as one sequencing sample. The sex of the pups was not determined before weaning (P0, P2, P5, P10, and P20). P56 male rats were used as a mature kidney control.

### Single cell isolation

For each sample, intact kidneys (from rats at postnatal days 0, 2, 5, 10, and 20) or ~3.0 g of kidney tissue containing both the cortex and medulla (rats at postnatal day 56) was minced into millimeter-sized pieces in DMEM/F12 (Gibco A4192002) containing 10% FBS (Gibco 10099141C). Then, the cells were digested in DMEM/F12 containing 1 mg/ml collagen type II (Sigma C6885) and 100 U/ml DNase I (Roche 4716728001) for 15–25 min at 37 °C with agitation. The samples were pipetted up and down every 5 min, and 20 µl samples were used to assess the digestion. After digestion, the fragments were filtered through a 40-mm nylon cell strainer (BD Falcon). The cell suspension was then centrifuged at 800 rpm for 5 min. After the removal of the supernatant, the cell pellets were washed three times with DPBS, resuspended in 5 ml of red blood cell lysis buffer (Miltenyi 130-094-183), and incubated for 2 min to remove red blood cells. Finally, the cells were resuspended by adding an appropriate volume of DPBS.

### Generation and sequencing of the 10x genomics RNA library

According to the manufacturer’s instructions for the 10X Genomics system, the cell suspension was input into a 10X Genomics Chromium device to capture 8000–12,000 cells. Then, barcoding and cDNA synthesis were performed, and a unique molecular identifier (UMI) was added to identify PCR duplicates. Thereafter, the samples were incubated with appropriate enzymes to produce full-length cDNA, and PCR amplification was conducted to generate sufficient quantities of material for library construction. The cDNA libraries were constructed and sequenced by Illumina NovaSeq.

### Data processing and analyses

After sequencing data were generated, quality control was first performed. For removal of potential doublets and low-quality cells, cells with <200 or >3000 unique expressed genes (as they are potential cell duplicates) were excluded, and genes expressed in ten or more cells were used for further analysis. Cells were also discarded if their mitochondrial gene percentages were over 70%. Finally, 25,400 genes across 54,704 cells were generated for further analysis.

After the application of quality control filters, the filtered gene-barcode matrix of all samples was integrated with Harmony to remove batch effects [67]. Then, the Seurat R package v3.1 was used for dimensionality reduction analysis. The normalized data function was used to divide the total number of reads in the cell, multiplying by a scale factor of 10,000 and taking the log2-transformed values. The top 2000 genes showing the greatest variability were then identified using the “vst” method under the Seurat Find Variable Features function, and these top 2000 genes were used to recover the same clusters. Then, we performed principal component analysis (PCA) using the variable genes as input, and 30 statistically significant principal components were applied for UMAP. The cells were clustered using the Find Clusters function, and clusters were generated by differential expression analysis (DEA). The clusters were viewed in UMAP clustering. The unsupervised clustering was performed at a resolution level of 0.5 and resulted in 25 clusters (Supplement Tables 1, 2 and Fig. S1). After identification by well-known cell type markers, the same cell types were combined, resulting in 13 clusters. Each cell type contains hundreds to thousands of cells indicating the number of biological repeats.

In the pseudotime trajectory analysis, the Monocle2 (version 2.10.0) algorithm was used to identify cell transitions. The major kidney cell types, namely, PDs, PTs, DCTs, LOH, CD-PCs, and CD-ICs, were used for pseudotime trajectory analysis. Additionally, significantly changed genes over the pseudotime were identified. Gene Ontology (GO) analysis was performed with clusterProfiler, which supports statistical analysis and visualization of functional profiles for genes and gene clusters.

## Supplementary information

supplement table 5

supplement table 6

supplemental material summary

supplementary Figures legends

Supplementary Figure 1(Figure S1)

Supplementary Figure 2(Figure S2).

Supplementary Figure 3(Figure S3).

Supplementary Figure 4(Figure S4).

Supplementary Figure 5(Figure S5).

supplement table 1

supplement table 2

supplement table 3

supplement table 4

## Data Availability

The datasets generated and/or analyzed during the current study are available in the Sequence Read Archive: PRJNA649702.
