# Supplementary material for: Determination of the dynamic cellular transcriptional profiles during kidney development from birth to maturity in rats by single-cell RNA sequencing
Source: Cell Death Discov. 2021 Jun 24;7:162. doi: 10.1038/s41420-021-00542-9 (PMC8257621; doi:10.1038/s41420-021-00542-9)
Supplement: Supplementary file 3 — supplemental material summary [file 41420_2021_542_MOESM3_ESM.docx]

**Supplementary information summary**

Supplementary information is available at (cell death discovery)’s website

Figure S1. 25 distinct cell clusters revealed by unsupervised clustering and shown in a two-dimensional UMAP map.

Figure S2. Verification marker genes in original cluster 4 by suing Kidney Cell Explorer Views

Figure S3. Identification of original cluster 21.

Figure S4. GO analysis (Cellular Component and Molecular Function) of top 200 DEGs of pseudotime path in each cell type.

Figure S5. Expression Features of known glomerular diseases genes.

Supplement Table 1. Median expression levels of each gene in original 25 clusters.

Supplement Table 2. Marker gene list of the original 25 original clusters.

Supplement Table 3. Marker gene list of Sub 9 clusters of original clusters 1 and 20.

Supplement Table 4. Gene list of the top 50 DEGs in pseudotime.

Supplement Table 5. Gene list of the top 200 DEGs of six major kidney cell types in pseudotime.

Supplement Table 6. GO terms of six major kidney cell types according to the top 200 DEGs in pseudotime.
