# Supplementary material for: Determination of the dynamic cellular transcriptional profiles during kidney development from birth to maturity in rats by single-cell RNA sequencing
Source: Cell Death Discov. 2021 Jun 24;7:162. doi: 10.1038/s41420-021-00542-9 (PMC8257621; doi:10.1038/s41420-021-00542-9)
Supplement: Supplementary file 4 — supplementary Figures legends [file 41420_2021_542_MOESM4_ESM.docx]

Supplementary Figure legends

Supplementary Figure 1(Figure S1). 25 distinct cell clusters revealed by unsupervised clustering and shown in a two-dimensional UMAP map.

(abbreviation: PT: Proximal Tubule, LOH: Loop of Henle, DCT: Distal Convoluted Tubule, CD-PC: Collecting Duct Principal Cell, PC1: Proliferating Cell 1, CD-IC: Collecting Duct Intercalated Cell, PD: Podocytes, SC: Stromal Cell, EC: Endothelial Cell, IC: Immune Cell, PC2: Proliferating Cell 2, UC: Urothelial Cell, ER: Erythroblast)

Supplementary Figure 2(Figure S2). Verification marker genes in original cluster 4 by suing Kidney Cell Explorer Views

(A-F) Heatmap of gene expression for top marker in original cluster 4 in Kidney Cell Explorer Views (https://cello.shinyapps.io/kidneycellexplorer/)

(G) Annotation of the number in the heatmap in (A-F).

Supplementary Figure 3(Figure S3). Identification of original cluster 21.

(A) uMAP plots of original 25 cell clusters in postnatal rat kidney.

(B-D) Gene expression plots for C1QTNF12, HAS2, WNT4

(E) Cluster hierarchy for indicating cluster similarities.

Supplementary Figure 4(Figure S4). GO analysis (Cellular Component and Molecular Function) of top 200 DEGs of pseudotime path in each cell type.

(abbreviation: GO: Gene Ontology, DEGs: Differentially Expressed Genes, CC: Cellular Component, MF: Molecular Function. PT: Proximal Tubule, LOH: Loop of Henle, DCT: Distal Convoluted Tubule, CD-PC: Collecting Duct Principal Cell, CD-IC: Collecting Duct Intercalated Cell, PD: Podocytes)

Supplementary Figure 5(Figure S5). Expression Features of known glomerular diseases genes.

Known glomerular diseases genes collected from OMIM databases were listed in all 13 cell type by heatmap. Each row represents one gene and each column is single cell type. Color keys represent the relative expression level.

(abbreviation: PT: Proximal Tubule, LOH: Loop of Henle, DCT: Distal Convoluted Tubule, CD-PC: Collecting Duct Principal Cell, PC1: Proliferating Cell 1, CD-IC: Collecting Duct Intercalated Cell, PD: Podocytes, SC: Stromal Cell, EC: Endothelial Cell, IC: Immune Cell, PC2: Proliferating Cell 2, UC: Urothelial Cell, ER: Erythroblast)
