# Supplementary figures and images for: Determination of the dynamic cellular transcriptional profiles during kidney development from birth to maturity in rats by single-cell RNA sequencing
Source: Cell Death Discov. 2021 Jun 24;7:162. doi: 10.1038/s41420-021-00542-9 (PMC8257621; doi:10.1038/s41420-021-00542-9)

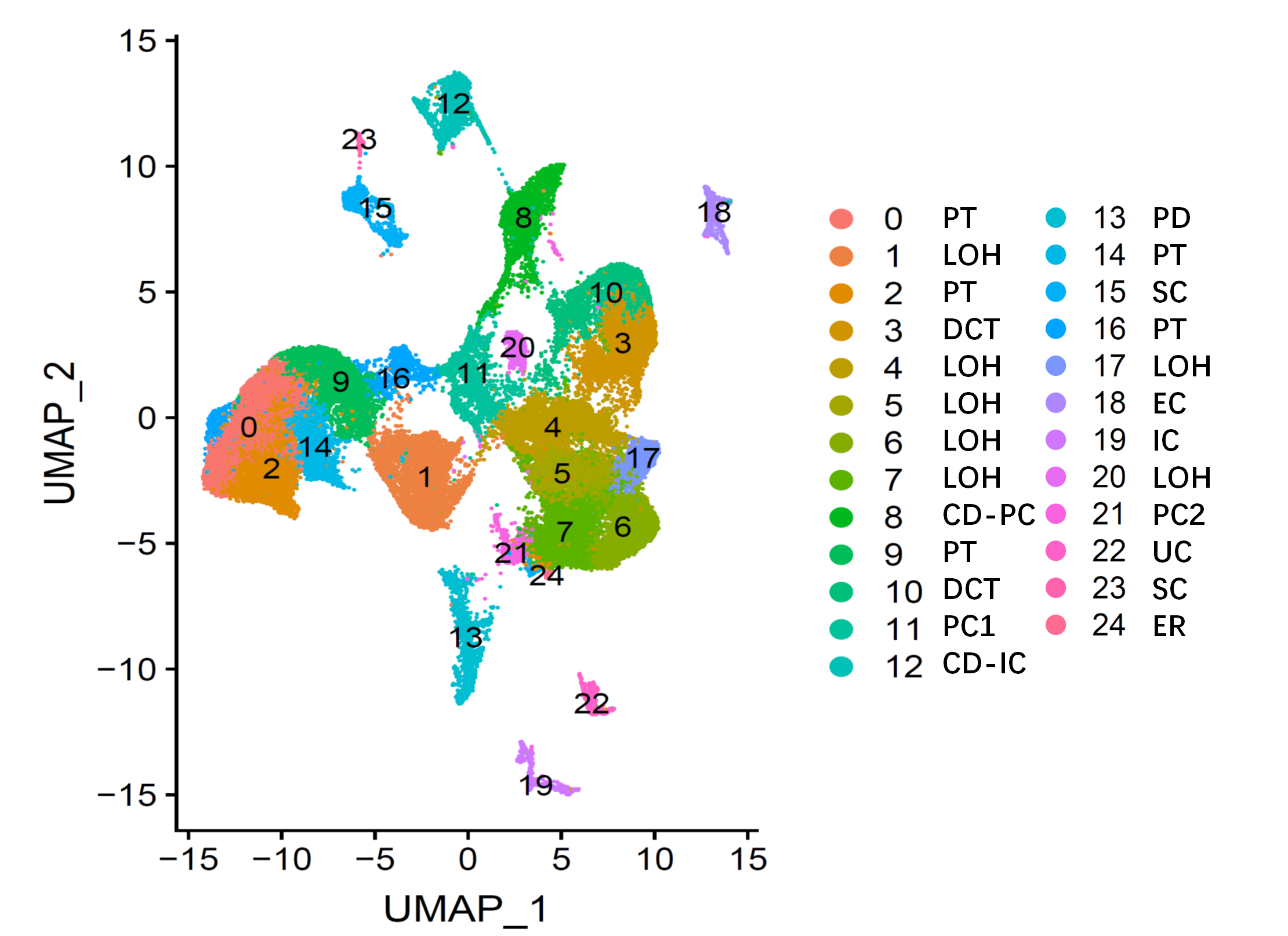

Supplement: Supplementary file 5 — Supplementary Figure 1(Figure S1) [file 41420_2021_542_MOESM5_ESM.tif]

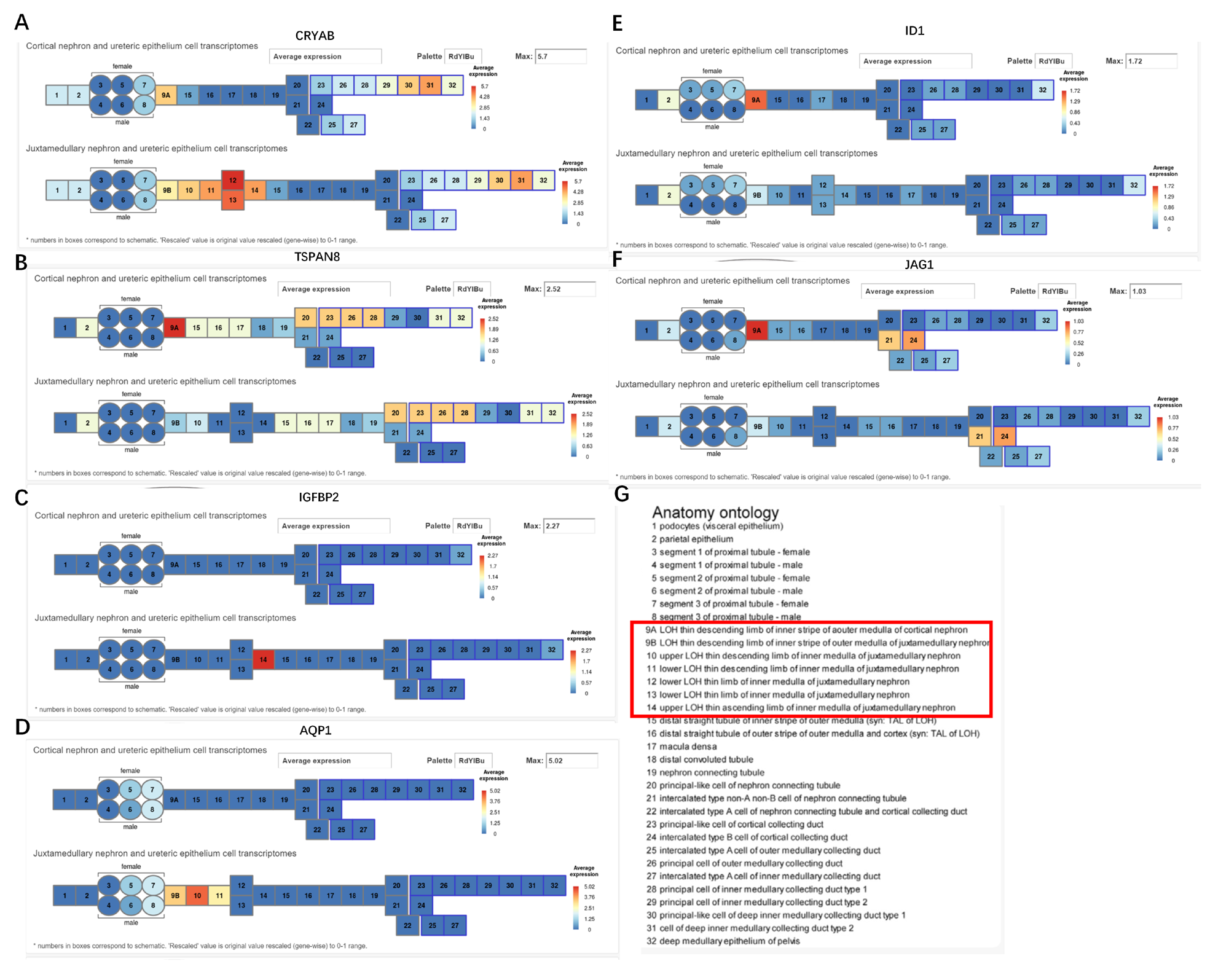

Supplement: Supplementary file 6 — Supplementary Figure 2(Figure S2). [file 41420_2021_542_MOESM6_ESM.tif]

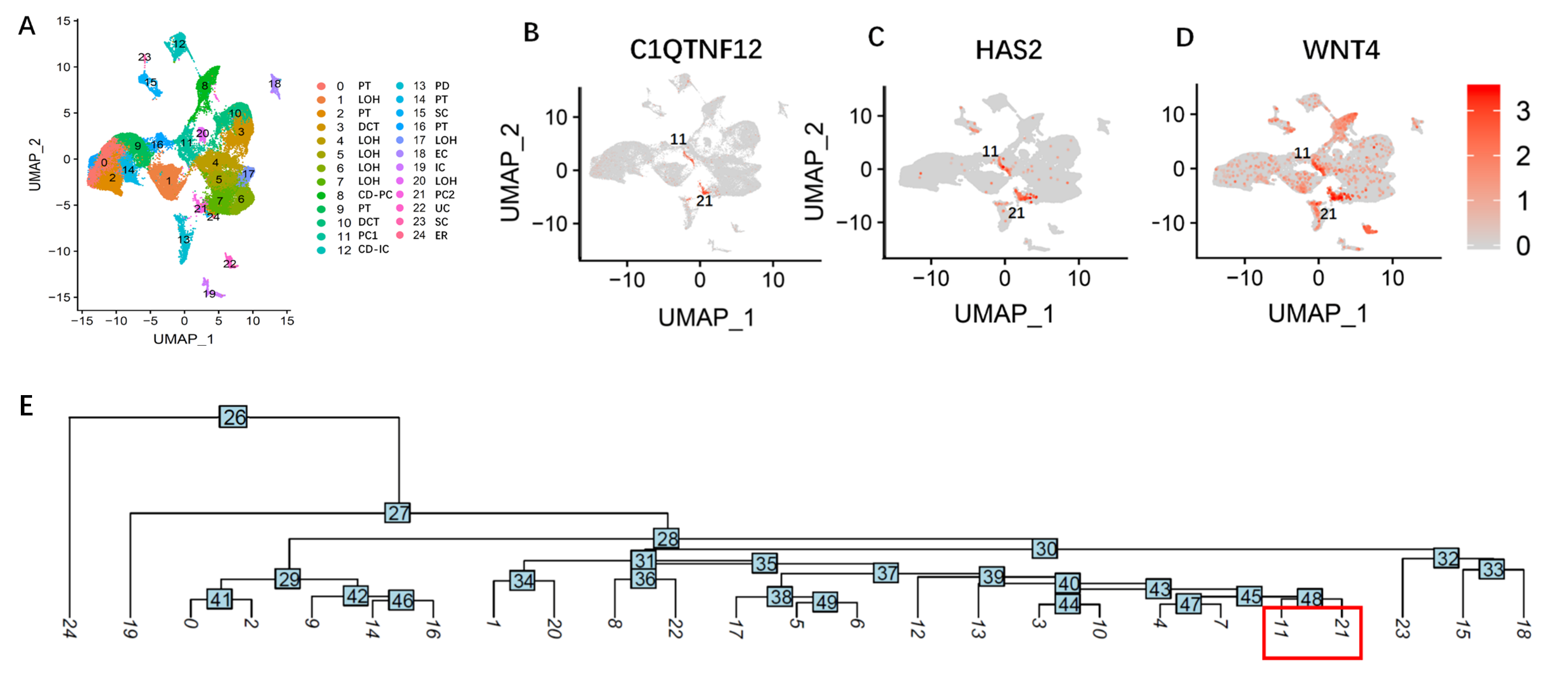

Supplement: Supplementary file 7 — Supplementary Figure 3(Figure S3). [file 41420_2021_542_MOESM7_ESM.tif]

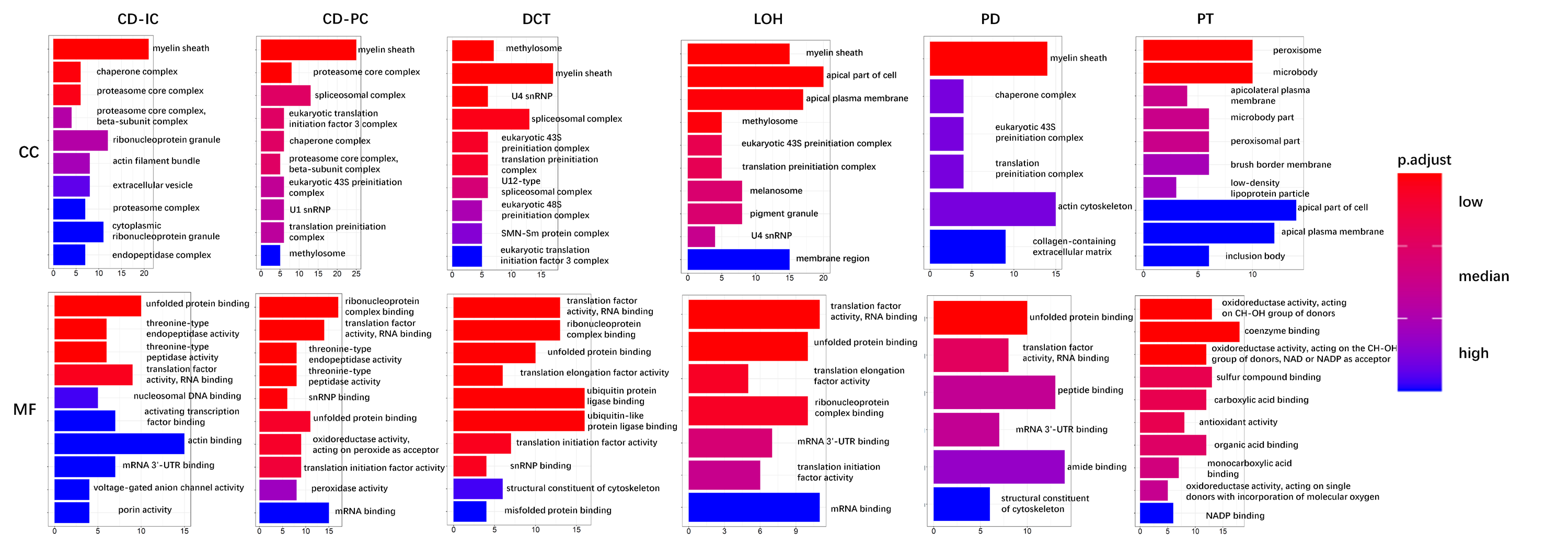

Supplement: Supplementary file 8 — Supplementary Figure 4(Figure S4). [file 41420_2021_542_MOESM8_ESM.tif]

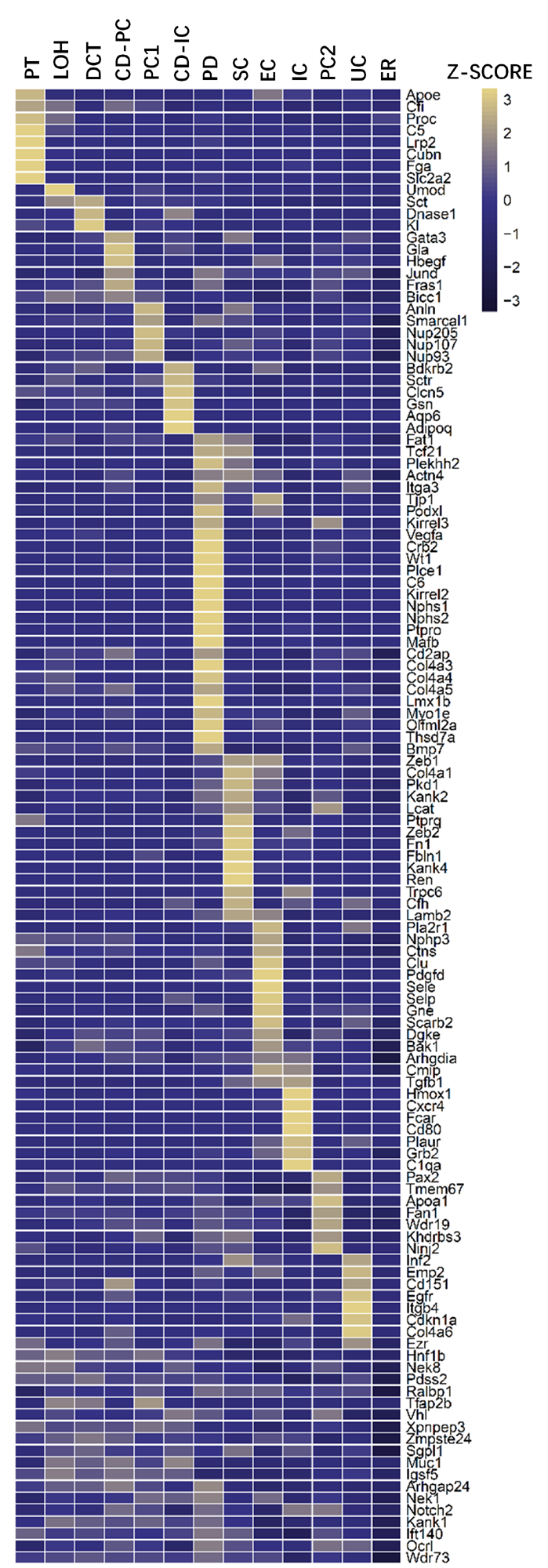

Supplement: Supplementary file 9 — Supplementary Figure 5(Figure S5). [file 41420_2021_542_MOESM9_ESM.tif]
